# Relationship of CK8/18 expression pattern to breast cancer immunohistochemical subtyping in Egyptian patients

**DOI:** 10.3332/ecancer.2014.404

**Published:** 2014-02-20

**Authors:** Hayam A Aiad, Rehab M Samaka, Nancy Y Asaad, Mona A Kandil, Mohamed A Shehata, Islam M Miligy

**Affiliations:** 1 Department of Pathology, Faculty of Medicine, Menoufia University, 32511, Egypt; 2 Department of Oncology, Faculty of Medicine, Menoufia University, 32511, Egypt

**Keywords:** CK8/18, Ki67 labelling index, immunohistochemical subtyping, breast carcinoma

## Abstract

The immunohistochemical (IHC) subtyping of breast cancer can be a useful substitute for gene expression analysis. The aim of this study was to investigate the relationship of CK8/18 to the biology of breast carcinoma (BC) represented by its IHC subtypes.

The IHC expression of CK8/18 was correlated with IHC subtypes of BC using ER, PR, HER2/neu, and Ki67 LI (with cutoff 14%). All cases showed CK 8/18 expression in tumour cells with varying degree of intensities; 49/70 cases (70%) showed diffuse cytoplasmic expression (loss of membranous pattern), while 21/70 cases (30%) showed membrano-cytoplasmic pattern. Adjacent non-neoplastic breast lobules showed membrano-cytoplasmic pattern in 58% of cases, which was significantly different from the pattern in invasive cancer (*P* = 0.002). A loss of membranous pattern in malignant tumours was significantly associated with higher tumour grade (*P* = 0.02), higher mitotic count (*P* = 0.03), and negative HER2/neu status (*P* = 0.04). CK 8/18 H score ranged between 1 and 290 with mean ± SD was 181 ± 70.54. Tumours with lower CK 8/18 H score were in the advanced stage group (*P* = 0.04). Low CK8/18 H score and loss of membranous pattern were significantly associated with triple negative (TN) subtype as compared with luminal subtype (*P* = 0.006 and *P* = 0.026, respectively). In addition, CK8/18 with lost membranous pattern was significantly associated with TN subtype compared with HER2/neu positive subtype (*P* = 0.001). However, there was no significant difference between luminal A and B subtypes regarding CK8/18 H score or pattern of expression. This study concluded that low CK8/18 H score and loss of membranous pattern of CK8/18 are associated with worse prognostic features and TN subtype.

## Introduction

Breast carcinoma (BC) in Egypt shows poor prognostic features, such as higher stage and higher grade than in developed countries, which may be attributed to different lifestyle and genetic factors [1].

Attention has been directed at molecular classification of breast cancer to stratify patients into well-defined prognostic categories that can be used in management decision [2, 3]. Standard immunohistochemical (IHC) tests (ER, PR, HER2, and Ki67) when used in a combinatorial manner can provide similar information as expensive molecular assays [4–6].

Keratins are epithelial-specific intermediate filament proteins, which are expressed in a tissue-specific manner [7]. CK18, a type I intermediate filament protein, and its co-expressed complementary type II partner, CK8, are persistently expressed in a variety of adult epithelial organs including breast and are also expressed by cancers that arise from these tissues [8]. However, regulatory changes in cytokeratins expression challenged the view that cytokeratins are only marker proteins [9]. Hypothetical mechanism for the role of CK18 in carcinogenesis states that CK18 acts as an identical target of Akt in the phosphoinositide 3-kinase pathway protecting cells from apoptosis [10, 11] and of extracellular signal-regulated kinase (ERK1/2) in the ERK mitogen-activated protein kinase pathway [12], and regulation of CK18 by Wnt is involved in Akt activation [13, 14].

This study was designed to evaluate the IHC expression of CK8/18 in BC of Egyptian patients to identify their relevance for IHC subtyping.

## Material and methods

This retrospective study was conducted on archival tissue from 70 BCs of Egyptian patients. The cases were diagnosed in the Pathology Department, Faculty of Medicine, Menoufia University, spanning the period between January 2007 and December 2010. Selection was based on the availability of paraffin-embedded blocks for serial cutting and examination.

### Histopathological assessment

Grading was performed according to the modified Bloom– Richardson method [15]. Scoring of mitosis was carried out using an Olympus CH2 light microscope with wide angle (field size: 0.274 mm^2^, field diameter: 0.59 mm). The mitotic figures were carefully defined to avoid inclusion of apoptotic cells. The mitotic activity index was calculated as the total number of figures counted in ten HPF fields of vision. The same cutoff as in previous publications was chosen, with ≥ 10 mitoses defined as high risk [16]. Nottingham prognostic index (NPI) is calculated according to Galea *et al* [17]. Grade I and II were lumped into one group for statistical purposes.

Staging was done based on the tumour node metastasis system [18]. For statistical purpose, cases were lumped into two groups: early stage—including stages I and II and advanced stage—including stages III and IV.

### IHC staining

IHC staining was performed using LAB-SA (labelled {strept} Avidin–Biotin) immunoenzymatic antigen detection system (Lab Vision/Neo Markers, California, United States). Antigen retrieval was done by boiling in citrate buffer saline (pH, 6), followed by cooling at room temperature. The primary antibodies were incubated overnight at room temperature. For CK 8/18, mouse monoclonal antibody keratin 8/18 Ab-2 ready-to-use was used (clone K8.8+ DC10; like 5D3, Lab Vision, Neo Marker). For Ki67, primary rabbit polyclonal anti-Ki67 antibody, MIB1 clone, M7240 was used and diluted 1:300 (DakoCytomation, Copenhagen, Denmark). Positive control slides were prepared by staining skin carcinoma (for CK 8/18) and tonsil (for Ki67). For oestrogen receptor (ER), primary antibody against ER was used (clone 1D5; Dilution, 1:50) (DakoCytomation). For progesterone receptor (PR), primary antibody against PR was used (clone IA6; Dilution, 1:50) (DakoCytomation). For HER2/neu: primary antibody against HER2/neu was used (clone 250, Dilution, 1:100) (DakoCytomation). BC cases previously known to be positive for ER, PR, and HER2/neu were used as positive control slides. Negative control slides were also included in each run and were done by the replacement of primary antibody by antibody diluents. Secondary antibody was applied with diaminobenzidine as a chromogen substrate and Mayer’s haematoxylin as a counter stain.

### Immunostaining interpretation

IHC staining of CK8/18 was evaluated in non-neoplastic and invasive cancer breast tissue concerning the pattern of expression either cytoplasmic, membranous or both [19]. H score was also calculated for CK8/18 using the intensity and percentage of positive cells [20]. The intensity score (0–3) was multiplied by the percentage of cells that stain with each level of intensity and the sum of these mathematical products was expressed as a score of 0–300. H score formula = strong intensity (3) × percentage + moderate intensity (2) × percentage + mild intensity (1) × percentage [20].

The Ki67 LI was determined using a semi-quantitative visual approach. Scoring was performed while blinded to patients’ information and outcomes. The entire slide was scanned for immunostaining evaluation using light microscope at low-power magnification (×100). All tumour cell nuclei with homogenous granular staining, multiple speckled staining or nucleolar staining were regarded as positively stained, regardless of intensity, while any cytoplasmic immunoreactivity was considered non-specific and hence not taken into consideration. Scoring was performed in the areas with highest number of positive nuclei (hot spot) within the invasive component of the tumour. The Ki67 LI (tumour growth fraction) was expressed as the percentage of Ki67-positive malignant cells among a total number of 1000 malignant cells, at high-power magnification (×400) [21]. Using 14% as an optimal cutoff point, cases were classified into low and high proliferative groups [22]. Histological grade II was also stratified into low and high proliferative subgroups: GIIa and GIIb [21].

ER and PR were considered positive if ≥ 1% of tumour cell nuclei are immunoreactive [23]. HER2/neu immunoreactivity was evaluated according to the American Society of Clinical Oncology guideline recommendations [24]. Positive HER2/neu cases were defined as 3+ positivity (>30% intense and complete staining); however, score 0 or 1+ was considered negative and 2+ cases were excluded.

According to the IHC results, ER, PR, HER2/neu, and Ki67 LI, we classified our cases into groups equivalent to molecular subtypes; luminal A subtype: ER+ and/or PR+, HER 2/neu−, with low Ki67 LI, luminal B1subtype: ER+ and/or PR+, HER 2/neu− with high Ki67 LI, luminal B2 subtype: ER+ and/or PR+, HER 2/neu + with any Ki67 LI, HER2/neu positive subtype: ER−, PR−, and HER2/neu positive, and finally triple negative (TN) subtype: ER− PR− HER 2 neu− [22].

### Statistical analysis

Data were statistically analysed using a personal computer with the Software Package for Statistical Analysis version 16 program; (USA). Chi-square test (*X*^2^ test) and Fisher’s exacts test were used to compare qualitative variables. Student *t*-test and Mann–Whitney (*U* test) were used to compare quantitative variables. Tests were considered statistically significant when (*P* ≤ 0.05) and highly significant when (*P* < 0.01).

## Results

The mean age of malignant cases was 49.97 ± 11.08, ranged between 25 and 81 years. The mean tumour size was 4.04 ± 1.95 ranged between 0.5 and 11 cm; 61/70 cases (87%) were invasive duct carcinoma of no special type (invasive carcinoma and no special type). The remaining cases were special types 9/70 (13%) including, four invasive lobular carcinoma, three medullary carcinoma, and two mixed tubular carcinomas.

Three out of 70 cases showed histological grade I (4%), 39/70 cases (56%) were grade II, and 28/70 cases were grade III (40%); 57/70 cases (81%) showed lymph node metastasis, 51/70(73%) cases showed advanced stage, 40/70 cases (57%) showed poor NPI, and 39/70 cases (56%) showed necrosis. The mean mitotic count was 5.56 ± 4.76 for all the cases ranged between 0 and 22. ER positive cases were 46/70 (66%), PR positive cases were 40/70 (57%), and HER2/neu positive cases were 22/70 (31%).

### CK8/18 immunostaining

**Figure 1.**
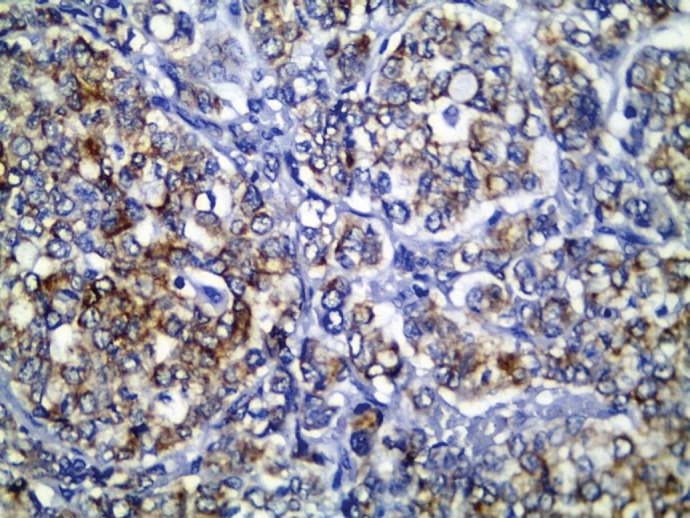
CK 8/18 cytoplasmic staining (loss of membranous pattern) in invasive duct carcinoma (immunoperoxidase ×400).

**Figure 2.**
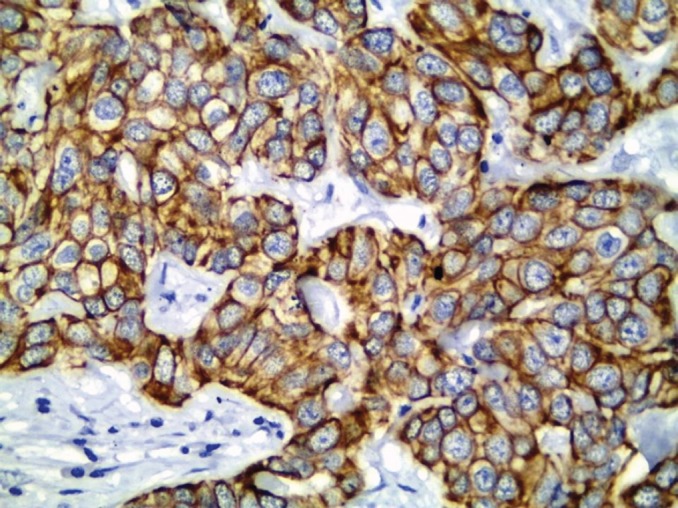
CK 8/18 with preserved membranous pattern in invasive duct carcinoma (immunoperoxidase ×400).

**Figure 3.**
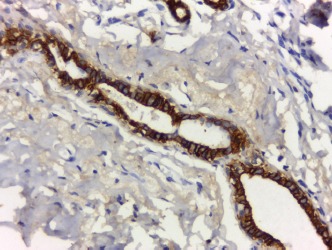
Non-neoplastic breast tissue shows membrano-cytoplasmic pattern (immunoperoxidase ×200).

All cases showed CK 8/18 expression in tumour cells with varying degree of intensities; 49/70 cases (70%) showed diffuse cytoplasmic expression (loss of membranous pattern; Figure 1), while 21/70 cases (30%) showed membrano-cytoplasmic pattern (Figure 2). Non-neoplastic breast tissue adjacent to invasive cancer was observed in 52 cases. Membrano-cytoplasmic pattern was observed in 30/52 (58%; Figure 3), which was significantly different from pattern in invasive cancer (*P* = 0.002). Loss of membranous pattern in malignant tumours was significantly associated with higher tumour grade (*P* = 0.02), higher mitotic count (*P* = 0.03), and negative HER2/neu status (*P* = 0.04; Table 1). CK 8/18 H score ranged between 1 and 290 with mean ± SD was 181 ± 70.54. Tumours with lower CK 8/18 H score belonged to the advanced stage group (*P* = 0.04; Table 2).

According to Ki67 LI, histological grade II was further stratified into low and high proliferative subgroups: GIIa comprised 17 cases (41.5%) and GIIb 24 cases (58.5). There was no significant difference between both groups regarding CK 8/18 pattern of expression or H score (Tables 1 and 2).

### IHC subtyping of carcinoma cases

Luminal A subtype represented 35 cases (50%), luminal B2 subtype: 11 cases (15.71%), HER2/neu positive subtype: 11 cases (15.71%), and TN subtype: 13 cases (18.58%). However, there was no case included in luminal B1 subtype. The luminal group was significantly associated with low histological grade, absent necrosis, and low mitotic count when compared with HER2/neu and TN groups.

### Comparison between different subtypes of BC cases, clinico-pathological parameters and CK8/18 IHC expression (Table 3)

High CK8/18 H score and preserved membranous pattern were significantly associated with luminal group as compared to TN group (*P* = 0.006 and *P* = 0.026, respectively). Luminal A subtype was significantly associated with more differentiated tumours (*P* = 0.03) and lower mitotic count (*P* = 0.05) when compared with luminal B subtype. However, CK8/18 H score or pattern of expression did not show a significant difference between both luminal groups.

There was no significant difference between HER2/neu and TN groups regarding the clinico-pathological features, Ki67 LI or CK8/18 H score. However, preserved membranous pattern of CK8/18 was significantly associated with HER2/neu positive group when compared with TN group (*P* = 0.001).

## Discussion

IHC subtyping of BC provides valuable information for clinical decision making. However, it is highly dependent on training, skills, and experience of laboratory personnel performing it. In addition variability in outcomes still present among patients of a particular subtype according to the current calcification system [25]. Therefore, there is an increasing need for additional refinement of prognostic factors to improve patient risk stratification. Cytokeratins have long been considered as markers for identifying epithelial origin [9]. In this study, we prove that CK8/18 has a strong relationship to biology of BC represented in its IHC subtypes.

In this study, CK 8/18 was expressed in all BC cases with varying degrees of intensity and percentage. Lower CK 8/18 H score showed significant association with advanced stage (*P* = 0.04). Similarly, low CK 8/18 expression in breast cancer cell lines was associated with high metastatic potential [26], and loss of these keratins was associated with a significantly worse prognosis [27]. The more interesting finding is the pattern of CK8/18 expression; we identified two main patterns of staining: membrano-cytoplasmic (30% of cases) and cytoplasmic patterns (70% of cases). We suggest a significant relationship between CK 8/18 pattern of expression and tumour behaviour, which may be explained by the subcellular localisation and the biological functions of CK 8/18. We claim that preserved membranous localisation (membrano-cytoplasmic pattern), which was significantly more observed in non-neoplastic breast lobules, may reflect a degree of differentiation that maintain the tissue integrity and resist stresses externally applied to the cell [28].

In contrast, we observed that loss of membranous pattern of CK 8/18 (cytoplasmic only) was significantly associated with higher tumour grade (*P* = 0.05), higher mean mitotic count (*P* = 0.033), and high proliferative group (*P* = 0.012). CK 8/18 is important for cellular processes, such as cell signalling [29], mitosis [30], cell cycle progression [31], and protection from apoptosis [10, 11]. Thus, it is involved in intracellular signalling pathways that lead to cell cycle progression which may explain the worse prognostic features associated with predominant cytoplasmic localisation with loss of membranous pattern of CK 8/18. Similarly, Cîmpean *et al* [19] observed three different distribution patterns of CK8/18 expression: diffuse cytoplasmic, membranous, and combined granular cytoplasmic with membranous but they did not correlate them to different clinico-pathological features.

One of the features common to BC is the increased rate of proliferation over that observed in normal breast epithelia [32]. It is now acknowledged that increased cell proliferation is a key determinant of poor outcome in patients with breast cancer [21, 33, 34].

Different cutoff points for Ki67 LI were tried to identify the valid prognostic one (10%, 14%, and 20%) [21, 22, 35]. In this study, we have assessed proliferative activity using Ki67 LI with the cutoff point of 14% because it is the best cutoff point to distinguish luminal B from luminal A tumours [22, 36].

Based on the IHC results of ER, PR, HER2/neu, and Ki67 LI, we classified our cases into four groups equivalent to molecular subtypes (luminal A, luminal B, HER2/neu positive, and TN subtypes). The IHC subtypes in the present series were comparable with the Western and Egyptian studies; 50% belonged to luminal A subtype in comparison with other studies: 41.2% [37], 53.1% [38], and 59.1% [39]. Luminal B subtype constituted 15.7% of cases compared with other studies: 13.9 % [37], 16.4 % [39], and 21.7% [38]. HER2/neu positive subtype constituted 15.71% compared with 8.5% [40], 12.7% [39], and 9% [38]. Finally, 18.58% were of TN subtype in comparison with 14.5% [40], 11.8% [39], and 16.2% [38]. 

As expected, the luminal subtype showed better clinico-pathological features when compared with HER2/neu and TN subtypes. We aimed to find out whether evaluation of CK8/18 is correlated to IHC classification of BC. CK8/18 has been called luminal marker as it indicates normal luminal epithelial-like differentiation [41]. CK 8/18 was expressed in all the studied BC cases including luminal and non-luminal subtypes. Our results indicate that the mere positivity of CK8/18 does not discriminate between luminal and non-luminal subtypes of BC. Therefore, we found that decreased CK8/18 H score and loss of membranous pattern was associated with TN subtype when compared with luminal and HER2/neu subtypes. These findings emphasise on the role of CK8/18 in the tumour biology of BC.

It has been suggested that luminal B subtype is equivalent to those that express either HER2/neu or Ki67 [35]. In this study luminal B was categorised as those showing Ki67 LI > 14% and all were found to be positive for HER2/neu coinciding with those called B2 subtype [22]. According to our results, the existence of B1 group, a subset of luminal B subtype, that shows negativity for HER2/neu in Egyptian population is questioned.

In this study, there was a statistical significant association between luminal B subtype and both higher tumour grade and higher mitotic count when compared with luminal A subtype. This coincides with poor prognosis associated with luminal B as compared with luminal A tumours [42–44]. However, there was no significant difference between luminal A and B subtypes with respect to CK8/18 expression.

There was no significant difference between the clinico-pathological features of HER2/neu and TN subtypes. In addition, there was no significant difference between both groups regarding Ki67 LI group. This agrees with previous reports that indicated that proliferation markers are of limited value in the TN and HER2/neu positive tumours as the majority of these tumours are poorly differentiated with a high proliferation index [45]. As regards to CK8/18 pattern of expression, loss of membranous pattern was significantly associated with TN group. This may further help in the differentiation between both groups since the treatment strategies differ.

The major limitation of this study is the small number of cases with available paraffin blocks suitable for recutting and immunostaining. This is because our hospital is a local centre with limited resources of archiving, documentation, and follow-up of cases. Moreover, in Egypt, we do not have a national wide program neither for breast cancer public awareness nor for screening. Therefore, cases are lately diagnosed and referred to the more equipped National Cancer Institute in Cairo. In spite of this limitation, our results indicate that the mere positivity of CK8/18 does not discriminate between luminal and non-luminal subtypes of BC; however, low CK8/18 H score and loss of membranous pattern of staining are associated with worse prognostic features and TN subtype.

## Conflicts of interest

No funding was received from any organisation.

Ethical approval was not required.

## Figures and Tables

**Table 1.** Association between CK 8/18 pattern of expression and the clinico-pathological parameters.

Variables	Loss of membranous staining N (%) 49 (70)	Preserved membranous staining N (%) 21 (30)	Test of significance	P value
*Age* Mean ± SD Median Range	50.89 ± 10.6148 25 – 81	47.80 ± 12.0852 30 – 68	*t* = 1.073	0.281
*Tumour size* Mean ± SD Median Range	4.01 ± 1.864 0.5 – 11	4.11 ± 2.204 1 – 10	*U* = 0.033	0.972
*Histological type* IDC (NST) Other types	42 (69) 7 (78)	19 (31) 2 (22)	Fisher’s exact test	0.714
*Grade* Grade I and II Grade III	28 (63.6) 21 (80.8)	16 (36.4) 5 (19.2)	*X*^2^ = 5.743	0.023^*^
*Lymph node status* Positive Negative	33 (58) 8 (61.5)	24 (42) 5 (38.5)	Fisher’s exact test	1.000
*Stage grouping* Early Advanced	14 (73.7) 35 (68.6)	5 (26.3) 16 (31.4)	*X*^2^ = 0.162	0.682
*NPI impact* Poor Intermediate Good	27 (67.5) 21 (80.2) 1 (25)	13 (32.5) 5 (19.8) 3 (75)	*X*^2^ = 5.412	0.070
*LVI* Present Absent	15 (71.7) 34 (69.4)	6 (28.3) 15 (30.6)	*X*^2^ = 0.022	0.862
*Necrosis* Present Absent	26 (66.7) 23 (74.2)	13 (33.3) 8 (25.8)	*X*^2^ = 0.462	0.493
*Mitotic count* Mean ± SD Median Range	6.28 ± 4.90 4 0 – 22	3.85 ± 3.99 2 0 – 12	*U* = 2.225	0.033^*^
*MAI* Low High	22 (47.8) 19 (79.2)	24 (52.2) 5 (20.8)	*X*^2^ = 6.384	0.012^*^
*ER status* Positive Negative	31 (67.4) 18 (75)	15 (32.6) 6 (25)	*X*^2^ = 0.437	0.511
*PR status* Positive Negative	28 (70) 21 (70)	12 (30) 9 (30)	*X*^2^ = 0.098	1.012
*HER2/neu status* Positive Negative	14 (63.6) 35 (72.9)	8 (36.4) 13 (27.1)	*X*^2^ = 0.614	0.043^*^
*Ki67 LI* Low proliferative rate High proliferative rate	18 (69) 31 (70)	8 (31) 13 (30)	*X*^2^ = 0.578	0.753
*Grading according to Ki67 (41 cases)* GIIa GIIb	9 (53%) 10 (42%)	8 (47%) 14 (58%)	*X*^2^ = 0.509	0.476

SD: standard deviation.

IDC: invasive carcinoma.

NST: no special type.

NPI: Nottingham prognostic index.

LVI: lympho-vascular invasion.

MAI: mitotic activity index.

ER: oestrogen receptor.

PR: progesterone receptor.

*X*^2^: Chi-square test.

*t*-Test: student T test.

*U*: Mann–Whitney *U* test.

* Significant.

**Table 2.** Association between CK 8/18 H score and different clinico-pathological features.

Variables	CK 8/18 H score Mean ± SD	Test of significance	*P* value
*Age group* ≤ 50 > 50	179.18 ± 78.00 182.12 ± 62.28	*U* = 0.035	0.973
*Tumour size* ≤ 2 > 2	155 ± 59 186 ± 72	*U* = 1.772	0.076
*Histological type* IDC (NST) Other types	187.04 ± 68.27 200.0 ± 18.25	U = 1.748	0.080
*Grade* Grade I and II Grade III	191.36 ± 63.48 162.30 ± 79.05	*U* = 1.334	0.184
*Lymph node* Positive Negative	182.2 ± 74.5 173 ± 50.8	*U* = 0.692	0.489
*Stage grouping* Early Advanced	188.03 ± 74.02 160.52 ± 57.20	*U* = 2.03	0.044^*^
*NPI impact* Poor Good and moderate	193.0 ± 77.23 164.0 ± 58.0	*t* = 1.772	0.088
*LVI* Present Absent	168.57 ± 61.34 185.71 ± 74.13	*U* = 1.102	0.275
*Necrosis* Present Absent	177.43 ± 73.18 184.51 ± 68.06	*U* = 0.153	0.874
*MAI* Low High	1.90 ± 6.46 1.62 ± 7.89	*U* = 1.252	0.211
*ER status* Positive Negative	193.91 ± 59.68 155.0 ± 83.19	*U* = 1.622	0.105
*PR status* Positive Negative	195.75 ± 58.69 160.33 ± 80.40	*U* = 1.513	0.136
*HER2/neu status* Positive Negative	185.0 ± 67.02 178.54 ± 72.69	*U* = 0.223	0.823
*Ki67 LI* Low proliferative rate High proliferative rate	197.30 ± 55.60 170.68 ± 76.92	*U* = 1.264	0.205
*Grading according to Ki67 (41 cases)* GIIa GIIb	181.76 ± 61.76 196.67 ± 68.75	*U* = 0.875	0.381

SD: standard deviation

IDC: invasive carcinoma.

NST: no special type.

NPI: Nottingham prognostic index.

LVI: lympho-vascular invasion.

MAI: mitotic activity index.

ER: oestrogen receptor.

PR: progesterone receptor.

GIIa: grade IIa.

GIIb: grade IIb.

*t*-Test: student *T* test.

*U*: Mann–Whitney *U* test.

* Significant.

**Table 3.** Multiple comparisons between immunohistochemical subtypes.

	Luminal A versus luminal B	Luminal versus HER2/neu	Luminal versus TN	HER2/neu versus TN
Age Mean ± SD	*U* = 0.492 *P* = 0.623	*U* = 1.682 *P* = 0.092	*t* = 1.169 *P* = 0.247	*U* = 0.843 *P* = 0.39
Size Mean ± SD	*U* = 0.374 *P* = 0.702	*U* = 0.561 *P* = 0.572	*t* = 0.862 *P* = 0.388	*U* = 0.401 *P* = 0.68
Histological type	*X*^2^ = 0.222 *P* = 0.893	*X*^2^ = 0.814 *P* = 0.664	Fisher exact *P* = 1.000	*X*^2^ = 0.882 *P* = 0.64
Histological grade	Fisher exact *P* = 0.032^*^	Fisher exact *P* = 0.007^**^	Fisher exact *P* = 0.0002^**^	Fisher exact *P* = 0.651
Lymph node status	Fisher exact *P* = 0.521	Fisher exact *P* = 1.000	Fisher exact *P* = 0.426	Fisher exact *P* = 0.576
Stage grouping	Fisher exact *P* = 0.293	Fisher exact *P* = 1.000	Fisher exact *P* = 0.090	Fisher exact *P* = 0.303
NPI	*X*^2^ = 5.48 *P* = 0.093	*X*^2^ = 0.303 *P* = 0.850	*X*^2^ = 2.452 *P* = 0.293	*X*^2^ = 3.025 *P* = 0.22
LVI	Fisher exact *P* = 0.123	Fisher exact *P* = 0.273	Fisher exact *P* = 0.734	Fisher exact *P* = 0.673
Necrosis	Fisher exact *P* = 0.083	*X*^2^ = 13.163 *P* < 0.001^**^	Fisher exact *P* = 0.026^*^	Fisher exact *P* = 0.223
Mitotic count Mean ± SD	*U* = 1.542 *P* = 0.052^*^	*U* = 2.063 *P* = 0.042^*^	Fisher exact *P* = 0.0004^**^	*U* = 0.903 *P* = 0.364
CK8/18 H score	*U* = 0.292 *P* = 0.762	*U* = 0.343 *P* = 0.733	Fisher exact *P* = 0.006^**^	*U* = 0.385 *P* = 0.703
CK8/18 pattern	*X*^2^ = 3.16 *P* = 0.203	*X*^2^ = 2.654 *P* = 0.264	Fisher exact *P* = 0.026^*^	*X*^2^ = 14.18 *P* = 0.001^**^
Ki67 proliferative group	—	Fisher exact *P* = 0.001^**^	Fisher exact *2P* = 0.003^**^	Fisher exact *P* = 1.000

SD: standard deviation.

NPI: Nottingham prognostic index.

LVI: lympho-vascular invasion.

* Significant.

** Highly significant.
